# Molecular Mapping of Antifungal Mechanisms Accessing Biomaterials and New Agents to Target Oral Candidiasis

**DOI:** 10.3390/ijms23147520

**Published:** 2022-07-07

**Authors:** Valentina Anuța, Marina-Theodora Talianu, Cristina-Elena Dinu-Pîrvu, Mihaela Violeta Ghica, Răzvan Mihai Prisada, Mădălina Georgiana Albu Kaya, Lăcrămioara Popa

**Affiliations:** 1 Department of Physical and Colloidal Chemistry, Faculty of Pharmacy, “Carol Davila” University of Medicine and Pharmacy, 6 Traian Vuia Str., 020950 Bucharest, Romania; valentina.anuta@umfcd.ro (V.A.); marina-theodora.talianu@drd.umfcd.ro (M.-T.T.); cristina.dinu@umfcd.ro (C.-E.D.-P.); razvan.prisada@umfcd.ro (R.M.P.); lacramioara.popa@umfcd.ro (L.P.); 2 Department of Collagen, Division Leather and Footwear Research Institute, National Research and Development Institute for Textiles and Leather, 93 Ion Minulescu Str., 031215 Bucharest, Romania; albu_mada@yahoo.com

**Keywords:** oral candidiasis, cellular response, epithelial damage, nanotechnological features, nanocolloids, inorganic materials, graphene oxide, polymers, vegetable resources, antifungal mechanisms

## Abstract

Oral candidiasis has a high rate of development, especially in immunocompromised patients. Immunosuppressive and cytotoxic therapies in hospitalized HIV and cancer patients are known to induce the poor management of adverse reactions, where local and systemic candidiasis become highly resistant to conventional antifungal therapy. The development of oral candidiasis is triggered by several mechanisms that determine oral epithelium imbalances, resulting in poor local defense and a delayed immune system response. As a result, pathogenic fungi colonies disseminate and form resistant biofilms, promoting serious challenges in initiating a proper therapeutic protocol. Hence, this study of the literature aimed to discuss possibilities and new trends through antifungal therapy for buccal drug administration. A large number of studies explored the antifungal activity of new agents or synergic components that may enhance the effect of classic drugs. It was of significant interest to find connections between smart biomaterials and their activity, to find molecular responses and mechanisms that can conquer the multidrug resistance of fungi strains, and to transpose them into a molecular map. Overall, attention is focused on the nanocolloids domain, nanoparticles, nanocomposite synthesis, and the design of polymeric platforms to satisfy sustained antifungal activity and high biocompatibility with the oral mucosa.

## 1. Introduction

Nowadays, oral disease management has become of significant concern in human health and is defined by serious disorders with a life-threatening pattern, affecting over 3.5 billion people worldwide, according to the World Health Organization [1]. Generally, tooth decay, periodontal disease, oral cancer, and oral candidiasis are important pathological conditions that warrant permanent attention and prevention. On the other hand, oral pathologies may trigger systemic diseases in the absence of care and prophylactic treatments. It is well known that tooth decay is directly related to the development of periodontal disease, both being linked to poor nutritional support for the patient [2]. Individuals suffering from periodontal disease are at high risk of developing severe cardiovascular events [3], heart attack, and stroke, which are responsible for a high mortality rate or health complications affecting the quality of life [4,5]. Furthermore, periodontal disease, characterized by chronic inflammatory events, was closely studied due to an additional risk of developing oral cancer in relation to habits such as smoking or alcohol intake [6]. In a cascade fashion, pathogenic microbial colonization in the oral cavity favors mycotic development and the occurrence of oral candidiasis.

The development of oral candidiasis is determined by several mechanisms that induce imbalances in the oral epithelium homeostasis. Poor local defense and a delayed immune system response are detrimental to cellular restoration. As a result, pathogenic fungal colonies disseminate and form resistant mixed biofilms by entailing bacterial pathogens and cellular fragments, promoting serious challenges in initiating a proper therapeutic protocol [7].

Multidrug resistance was established as a serious concern that creates unsuccessful results following treatment with classic topical drugs, particularly polyenes and azoles. Enzymes’ structural modifications, efflux pumps, gene regulation, and mutations are the main mechanistic pathways of *Candida* sp. implied in drug resistance [8]. One of the main components that arouse interest in studying new antifungal agents is the fungi cell wall. The cell wall represents the indwelling place of key proteins implied in gene regulation and related to adhesion, dissemination, and immune response [9]. Hence, this study of the literature aimed to discuss possibilities and new trends through antifungal therapy for the buccal route. Many studies have explored the antifungal activity of new agents or synergic components that may enhance the effect of classic drugs by targeting molecular levels. 

The most studied mechanisms related to the antifungal activity proposed the modification of the cell wall structure, the passage through the inner layers, triggering alterations in the cytoplasmic membrane, and cellular death. It was found that these events can be connected with the presence of genuine surface stabilizers selected in nanocolloids development, particularly micro-/nanoemulsions [10]. Incorporating poorly water-soluble compounds in the formulation process of nanoparticulate agents can improve drug passage through the oral tissue, permitting targeted action in the affected site [11]. Inorganic nanoparticles synthesized from various natural resources represent new biomaterials that can be adequately processed to promote oxidative stress and fungi death or provide a gene regulation capacity without inducing cytotoxicity in healthy cells [12,13]. Combining nanoparticles with organic graphene-oxide-based scaffolds or polymeric supports defines a new beginning to developing smart biomaterials for delivering antifungals such as clotrimazole [14] or miconazole [15], the most selected azoles for oral applications. 

Overall, attention is focused on the nanocolloids domain, nanoparticles, nanocomposite synthesis, and the design of polymeric platforms to satisfy sustained antifungal activity and high biocompatibility with the oral mucosa. The assessment of molecular targets in the biomaterials domain is considered by creating a molecular map with the most valuable antifungal agents. 

## 2. Oral Health and Candidiasis Development

Oral fungal infection is one of the most researched medical challenges today, being critically related to the development of oral candidiasis, the dissemination of *Candida* sp. in oromucosal tissues, overcoming barriers such as antifungal drug resistance, and repurposing new pathways and mechanisms to alleviate resilient infections. The discovery of new targeted therapeutic agents exhibiting intelligent mechanisms of action remains of major concern, especially in the case of chronic and immunocompromised patients [16,17].

As a part of the gastrointestinal tract, the oral cavity is usually a pleasant environment for commensal microorganisms in the absence of health imbalances [18]. Mycotic infection of the oral mucosa acts as an invasive pathology that involves the development of opportunistic pathogens in the structure of the healthy tissue of the host. Depending on the predisposing factors, the presence of systemic disease, and the status of the immune system, infection with *Candida* can become life-threatening, with a reserved prognosis and a high mortality rate of 71–79%, especially in patients with indwelling catheters or following immunosuppressive treatments [16,19]. 

Infection with *Candida albicans* is one of the most encountered types of oral mycosis in 95% of cases [20] and is known usually as oral candidiasis, oropharyngeal candidiasis, or oral thrush [21], affecting not only the extremes of ages—neonates and elderly individuals—but also the adult population with comorbidities [16]. Its development leads to the formation of white patterns with a cotton-like feeling at the level of the epithelium of the mouth and throat, accompanied by inflammatory lesions, discomfort, and limitations in swallowing [22]. Other species reported in the literature as promotors for candidiasis occurrence are *C. glabrata*, *C. tropicalis*, *C. pseudotropicalis*, *C. krusei*, *C. lusitaniae*, *C. dubliniensis*, *C. parapsilosis*, or *C. stellatoidea* [16,20]. In severe cases related to immunocompromised diseases, pathogens such as *Cryptococcus neoformans* were found to produce unexpected tongue lesions in an HIV patient [23], while mucormycosis caused by mucormycetes was recently reported with a high incidence in COVID-19 patients [24,25], along with candidiasis [26]. The newly developed infections that occurred in the new context of the COVID-19 pandemic were analyzed as multifactorial cases, and an immediate action was taken to combat fungi resistance and avoid the death of hospitalized patients. 

Hence, it can be appreciated that the development of oral candidiasis is influenced by several local and systemic factors, which will be briefly pointed out, as shown in Table 1. Thus, the main local triggers involved in oral candidiasis pathogenesis are related to the presence of imbalances in the normal salivary flow [27], the use of dentures or dental prosthesis [28,29], diet factors [30], poor local defense at the epithelial level [31], oral dysbiosis [32,33], the use of inhalator corticosteroids [34], smoking [35], or the presence of other local oral lesions which predispose the oral mucosa to abnormal changes [36]. On the other side, the influence of a series of systemic factors such as deficiencies in the normal levels of vitamins and minerals [37], the presence of metabolic disorders [38], menopause [39], HIV infection [40], the completion of prolonged antibiotherapy [21] and immunosuppressive treatments, especially related to weakened immunity in cancer patients who followed chemotherapy [41] and radiotherapy of the head and neck [42], and not least those who suffered from COVID-19 and associated comorbidities [26], may induce the development of chronic candidiasis, which is hard to control with conventional therapy. 

The development of candidiasis as a result of cytotoxic treatments during chemotherapy and radiotherapy represents a long-term concern that remains one of the main priorities in the palliative care of cancer patients [41]. There is well-established statistical coverage for candidiasis prevalence worldwide [22]. Still, cancerous patients have one of the highest predispositions to develop a chronic fungal infection during therapy, after HIV patients [16]. In earlier reports, according to Akpan and Morgan, the oral carriage of *C. albicans* related to chemotherapy in acute leukemia was ~90% [19]. In a recent study by Aslani N. et al., *C. albicans* was encountered as a pathogenic strain in 50.6% of cases from the group of 350 cancer patients included in their study [41]. In the new era of smart therapies, the research of innovative drug delivery systems leads to knowledge of the mechanism of fungi invasion in healthy epithelial structures, the molecular background of the infection to overcome the resistance of fungal strains to conventional therapies, and the recovery of epithelial poise.

*C. albicans* may exist in four classic states—yeast, pseudohyphae, hyphae, and chlamydospores, characterized by dynamic morphological switching. However, up to nine morphologies have been described to date, of which the opaque (a/α), grey, and gastrointestinally induced transition yeast-like cells were studied for their behavior in the process of pathogenesis [59].

*C. albicans* infects the host by two primary mechanisms: specifically, an induction of the receptor-mediated epithelial cell endocytosis, or active penetration [60]. Other mechanisms were previously described, with specific attention paid to the passage of *C. albicans* in the presence of Flu1 efflux pumps. The efflux pumps protect *C. albicans* by host defense peptides via SAPs. Subsequently, proteolytic degradation of the intercellular junctions sustains *C. albicans* passage and completes its invasive character. The affinity of the fungi for bacterial entities and the secretion of toxin-like candidalysin determine the high virulence and an enhanced capacity to invade the epithelial tissue [61]. However, in the absence of triggers, *C. albicans* inhabits the lining mucosa, along with other microorganisms, as part of commensal microflora in a calm and friendly environment.

Thereby, in a normal state, the oral mucosa acts as a physiological barrier against physical and chemical agents due to its well-arranged epithelial structure, which forms the entry lining of the gastrointestinal tract [62]. As well as in the case of skin structure, oral mucosa plays a major role as an immunological and microbiological defense gate, but its structural peculiarities create a unique environment imagined as a mosaic of cellular arrangements, a key factor in the oral pathogenesis of candidiasis, the process of response to infection, and drug delivery [62,63]. The oral mucosa has a thickness of 500–800 μm, covering the entire surface of the oral cavity. It is a stratified, non-keratinized epithelium connected with flexible connective tissue, specific to the buccal, soft palate, and sublingual mucosa. Then, the mucosa is continued with a stratified, keratinized epithelium in the hard palate and gingival areas, being linked with collagenous connective tissue as the intimate lining in the proximity of maxillofacial bone tissues [64]. 

At the epithelium level, cells are connected by tight junctions, gap junctions, and anchoring junctions, providing integrity for the entire lining [62]. A rapid epithelial clearance, along with salivary flow produced by salivary glands, presents a protective function to avoid the adhesion and invasion of microorganisms [65]. Moreover, the barrier effect is strengthened by the presence of nanometric membrane granules which release lipidic compounds in the intercellular spaces [66]. From an in-depth perspective, the lipidic component contains a high proportion of phospholipids [67], ceramides, and small levels of cholesterol sulfate and glycerol ceramides [64].

As exemplified in Table 1, several factors, such as cytotoxic agents, radiation, and immunosuppressants, provoke local imbalances in the normal homeostasis of the epithelia, followed by the destabilization of cell arrangement, triggering a cascade of immunological reactions. This event acts in favor of *C. albicans* pathogenesis and invasion, from local development through systemic disease [68]. Lesions occurring in the oral mucosa and the gastrointestinal tract may determine the dissemination of the fungi in organs such as the lungs, liver, kidney, spleen, or brain [69]. In severely immunocompromised patients receiving chemotherapy and in the absence of appropriate treatment, it was stated that oral candidiasis can expand its development through the pharynx and esophagus, reaching the bloodstream by accessing the intestinal epithelium [70,71].

*C. albicans* is characterized by smart structural behavior. This fact represents the basis of antifungal drug resistance and its ability to interact with the host immune system and invade healthy tissues. Here, the most important mechanisms related to the multidrug resistance and the narrowing of treatment options will be briefly presented, taking into consideration *C. albicans* morphology.

*C. albicans* grows as yeast—in round to oval structures with an important role in adhesion—and as pseudohyphae or hyphae structures—tubular cells with filamentous patterns [72]. Their development is quite sensitive to ambient factors, specifically body temperature, local pH, salivary flow, human serum, CO_2_/O_2_ tension, and the presence of commensal Gram-positive and Gram-negative bacterial species [44,73]. This symbiotic relationship offers advantages for both microbial entities, capitalizing on the formation of polymicrobial films [74]. To exemplify, streptococci offer a carbon substrate from lactate excretion for *C. albicans* development, while in reverse, the fungal colonies assure a reduced oxygen tension for living bacteria [21,75]. *Streptococcus mitis*, *S. oralis*, and *S. gordoni* interact with *C. albicans* by cell surface proteins, which have an affinity for the cell wall of the fungus containing adhesins [75]. Furthermore, *Staphylococcus aureus* has an attraction to the hyphal structure of *C. albicans*, and by default, their interactions increase the virulence of the biofilm [74]. 

As a mechanistic pathway, the transition through the hyphal state is mediated by multiple transcriptor factors via gene regulation [73]. Under ambient stimuli, *C. albicans* will express hypha-specific genes in numerous pathways, namely: the cAMP/PKA primary pathway mediated by the stimulation of CO_2_ and serum, the chaperone heat shock protein 90 (Hsp90) pathway mediated by temperature, the Rim 101 pathway, specific under the effect of acidic pH [76], and, in the last case, the pescadillo homolog (Pes 1) pathway, responsible for hyphal to yeast inversion [76,77].

**Figure 1:**
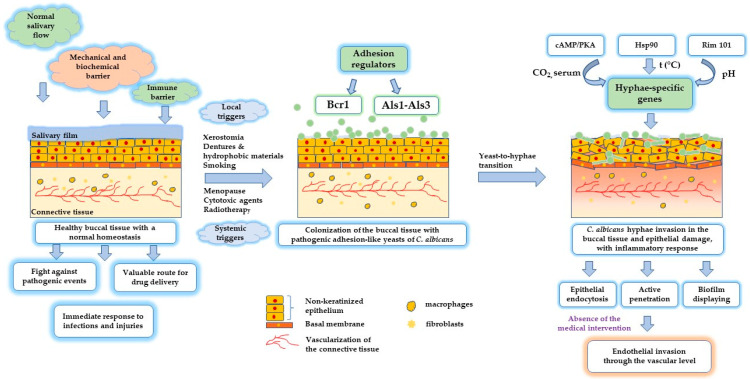
Dynamics in the colonization process of the oral mucosa with *C. albicans* and molecular processes, triggering yeast to hyphae transition and invasion.

Considering the previously detailed aspects, a concise presentation of the main processes implied in oral candidiasis pathogenesis is rendered and described in Figure 1. The emphasis is on the impairment to normal homeostasis and the implications of the adhesion regulators for *C. albicans* yeast-like colonization, followed by changes in the oral epithelium under the effect of hyphae invasion. 

**Figure 2:**
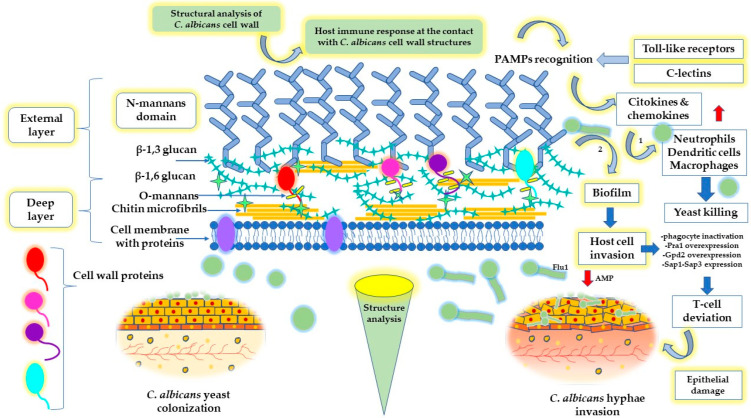
Cell wall structure and host immune defense in *C. albicans* epithelial infection.

The bi-layered cell wall represents a fundamental structure that assures the protection and adhesion of the fungi at the level of the epithelial host cells. Its composition, based on immunogenic elements such as chitin, β (1–3) glucan, β (1–6) glucan, mannan, and mannoproteins, creates an interface to interact with the host immune cells via glycoconjugates, representing the main target of antifungal drugs from the echinocandins’ class [78]. The cell wall provides protection against shocks, having a rigid structure [79]. On the other side, elasticity and flexibility were found to be part of the adaptable nature of the fungi to modulate the tensile strength in response to environmental stress conditions such as osmotic shocks [79,80]. Several methods were proposed to study the cell wall’s morphology and chemical structure. Lenardon, M.D. et al. offered a valuable interpretation by appealing to 3D modeling and molecular scaling [81]. Thus, in the inner layer of the cell wall, chitin microfibrils coexist with the cell wall proteins, providing vital activity for fungi growth. Chitin microfibrils are crossed by β (1–3) and β (1–6) glucan helices and then interconnected with the cell wall proteins through covalent bonds [81,82]. N-mannan structures define the outer layer of the cell wall as a fibrillar layer and bind the proteins positioned on the inner side. Glycosylphosphatidylinositol proteins, defined as Als3, Als9-2, Sod5, Sap1, Sap3, endoglucanase, exoglucanase, and chitinase, are two sides grafted with both N-mannan and O-mannans and covalently attached to chitin microfibrils by β (1–6) glucan. Pir proteins are attached to N-mannans and covalently linked to an alkali-sensitive bond. The cell membrane integrates proteins with catalytic and transport activity [78,81]. At this level, lipids continue to build the cellular membrane of fungi, promoting structural integrity and protection. Cell morphology, the yeast to hyphae transition, and the biofilm dynamics depend on plasma membrane composition, specifically sterol, sphingolipid, and phospholipid content, which is of paramount importance for the adhesion process and fungi resistance. The higher the polar lipid level in the cell membrane, the better the consolidation of the biofilm will be [83]. A graphical interpretation of the cell wall structure of *C. albicans* is presented in Figure 2, with attention paid to the main targets of the host immune defense.

The adhesion process of the yeasts at the level of epithelial surfaces or abiotic areas of dental biomaterials with a hydrophobic nature is strengthened by the biofilm generation displayed in the presence of protein-based adhesion regulators, namely Bcr1 via Hwp1 gene [84], Als1 and Als3 [7,85]. This process is continued with the development of hyphal cells, which contribute with a series of regulator factors known as Efg1, Tec1, Ndt1, and Rob1 to consolidate the biofilm [86], create a powerful barrier against host immune system intervention, and disseminate by building a favorable environment for the proliferation of mixed biofilms in the presence of other microbial species [7]. In the maturation process of the biofilm, an expansion by the extracellular matrix was observed, and the engulfment of singular structures such as yeasts, pseudohyphae, hyphae, conglomerates containing *C. albicans* lysed particles after the action of the immune system, host cells, erythrocytes, epithelial cells, dead cells, neutrophils and polysaccharides [87,88]. The proliferation of *C. albicans* biofilm is continued with the dispersion of yeast-like cells under the effect of transcriptional regulators such as Nrg1, Ume6 [89], Pes1 [83], Hsp90 [90], and cell wall protein Ywp1 [91], in a similar manner to that of the planktonic phase, but exhibiting a high virulence [7].

The destructuration of the oral epithelium represents a sign of symptomatic candidiasis, marked by a rapid expansion through the use of extracellular matrix and blood constituents to promote dissemination and invasion [60]. The polymorphism represents the ability of *C. albicans* to pass from an adhesion-like state of yeast—implied in the attachment at the level of the epithelial cells—through an intermediate pseudohyphal and filamentous hyphal-based morphology [21,60]. The hyphal form invades the epithelium by passing the tight junctions and creating depressions when active penetration occurs, or membrane ripples and protrusions, particularly for endocytosis, as was emphasized in the TEM and SEM microscopic analysis of Wächtler, B. et al., and Dalle, F. et al. [60,92]. This process is mediated by the lytic enzymes (proteases) and invasins secreted by hyphal cells, which bind and degrade E-cadherin and other structural proteins implied in cell stability and their interaction. Agglutinin-like sequence-3 (Als3) represents one of the invasins implied in endocytosis [93], along with the heat shock protein Ssa1 from the Hsp70 family [90,94].

The immunological pattern of *C. albicans* has for years opened a broad area of research intending to propose specific mechanisms implied in growing fungi cells, discovering their vulnerabilities. According to previous reports, important mechanisms that create a cycle of fungi invasion and interaction with the host immune cellular pathways will be further described. At the contact with the antigen, the innate immune system initiates a defense against the pathogens. Phagocytes are activated and will recognize the pathogen-associated molecular patterns through the pattern-recognition receptor pathways (PAMPs) [7,95], followed by signal-mediated transcription. These PAMPs are the structural elements of the cell wall expressed as N-mannans and β-glucans, easily identified by the C-lectins and Toll-like receptors of the host [95]. In this way, the immune system’s inflammatory response will be quantified by an increased level of cytokines and chemokines. An increase in the level of neutrophils, macrophages, and dendritic cells is favorable for killing the pathogen and activating the adaptive immune response [7]. The immune response is higher in the yeast form and reduced in the hyphal state, where the transcriptional regulators of the cell wall proteins will act in the sense of host cell invasion [96]. The adaptive immune system releases antibodies to counteract PAMPs activity. On the other hand, the success of an immediate reaction of the host immune system is limited in the case of biofilm attachment, evolving resistance, and a higher virulence, which results in the underestimated activity of neutrophils [97]. Besides the penetration activity, hyphal cells sustain the inactivation of the immune response by destroying phagocytes [7]. In the biofilm state, the overexpression of pH-regulated antigen 1 (Pra1) and glycerol-3-phosphate dehydrogenase 2 (Gpd2), together with the aspartyl proteinases Sap1-Sap3 activity, sustain complement inactivation. Pra1 protein acts in multiple ways, favoring *C. albicans* infection. As a pH-dependent zincophore, it attaches to the immune cells (e.g., CD4+ T cells) and deviates their action to reduce the cytokine cascade. Gpd2 targets the epithelial and endothelial cells, while Sap1-Sap3 attaches complement proteins and degrades them via proteolytic reactions [98]. Subsequently, it was found that Msb2 glycoprotein degrades the AMP, while Sap’s degradative injuries assure the release of nutrients that sustain *C. albicans* development [99]. The mechanism was proposed as the cleavage of Msb2 under the action of Saps. Consequently, the extracellular domain of Msb2 is released in the invasive medium of fungal cells. However, this complex inactivates human cathelicidin LL-37, histatin-5, hNP-1, and hBD-1. Similarly, polyamine efflux transporter Flu1 reduces AMP activity through the efflux of histatin-5 [99,100]. 

On the other side, another essential component of the oral mucosa sustains the immune cells’ defense. The mucus generated by salivary activity promotes protection against pathogens due to its composition based on mucins [44]. In the study of Kavanaugh, N.L. et al. mucins were evaluated as potent glycopolymers to decrease fungi virulence. It was discovered that the coded MUC2, MUC5AC, and MUC5B mucins suppress *C. albicans* virulence related to hyphal activity, suppress filamentation, and inhibit adhesion [101]. Generally, the response of innate immunity can be increased with several defense proteins identified in the saliva. Besides mucins, lysozymes, statherins, cystatins, or proline-rich proteins act synergistically to counteract tissue imbalances. Salivary immunoglobulin and salivary chaperonin stimulate the activity of both innate and acquired immunity [65]. 

In a pathological state, when the immune response is weakened, the invasive mechanisms of *C. albicans* favor the epithelial damage and the development of acute, chronic, or chronic mucocutaneous candidiasis [21]. Acute candidiasis is characterized by pseudomembranous and erythematous forms, being specific for chronic conditions as well. Pseudomembranous infection is commonly recognized as a complex of damaged epithelial cells and necrotic tissue braided with hyphal cells in a plaque-like architecture. After debridement, a bleeding tissue is disclosed [102]. More frequently encountered after using broad-spectrum antibiotics, acute erythematous candidiasis or atrophic candidiasis is perceived in the form of acute painful and swollen oral lesions with a red appearance on the dorsum of the tongue and palate. For patients wearing dentures or orthodontic retainers, chronic atrophic candidiasis (denture stomatitis) usually occurs [103]. Likewise, a complex of stable non-detachable plaque type and erythematous lesions with nodular appearance better describes the chronic development of hyperplastic candidiasis on the oral mucosa and lateral area of the tongue. These patients are at risk of developing epithelial dysplasia and cancerous lesions [104]. Other lesions associated with *C. albicans* development target the commissures of the lips in angular cheilitis or the dorsum area of the tongue, where symmetrical disposal of the lesions determines the shaping of median rhomboid glossitis. The lesions can appear independently or in mixed pathologies associated with pseudomembranous and erythematous candidiasis and constitute a real challenge for an appropriate therapeutic purpose [105].

## 3. Antifungal Therapy and Multidrug Resistance

Many attempts have been proposed to offer valuable therapeutic solutions for the treatment of oral candidiasis. Local therapy remains essential for the chronic management of fungal lesions. Additionally, here, nystatin as a suspension or tablets, clotrimazole lozenges, miconazole-based gel, or ketoconazole in the form of gel, tablets, or a suspension are the standard choices to alleviate and treat superficial infections [106]. A decreased bioavailability of drug molecules characterizes the use of conventional formulations poorly tailored for buccal administration through the affected tissue. However, the oral tissue dynamics, expressed by a permanent salivary activity, enzymatic activity, and the barrier effect of the epithelium [64], determine deficiencies in the retention and penetration of antifungals, especially for active substances with a high molecular weight. In addition, the fungal cell resistance, mediated through multiple mechanisms, rallies drug activity, and the formation of biofilms sustains the generation of numerous resistant strains [8], as exemplified in Table 2. In this case, the initiation of systemic treatment can avoid fungi dissemination. However, the smart modulation and versatility of *C. albicans* cells create real challenges in both the topical and systemic delivery of antifungals.

Nystatin represents a standard in the local therapy of oral candidiasis, along with amphotericin B, which is kept as a second therapeutic choice for systemic use, due to its nephrotoxic character. Polyene macrolides have an affinity for ergosterol and exert fungicidal action by disrupting the cellular membrane of the fungi [106,107]. Nystatin suspension in doses of 100,000 IU/mL, four times a day, remains a classic approach for local applications [106]. Beginning with the need to reduce the local discomfort, the undesirable taste, the poor retention, and solubility problems, nystatin was incorporated into nanoemulsion-based vehicles, which can enhance local penetration through the mucosa and antifungal activity on *C. albicans* with a reduced minimum inhibitory concentration (MIC) of 0.78 μg/mL [108]. In the case of amphotericin B, new solutions were proposed to attenuate its toxicity and improve pharmacological assets. Liposomal formulations available on the market under the name of AmBisome^®^ use phospholipidic envelopes, combined with distearoylphosphatidyl glycerol and cholesterol, to retain amphotericin B and deliver it through the ergosterol sites in a targeted manner [109].

As the main representatives of the azoles class preferred for buccal delivery, clotrimazole and miconazole are imidazole compounds that inhibit the 14-α-demethylase enzyme implied in ergosterol synthesis [20]. As an efficient alternative to clotrimazole lozenges, 10 mg, administered up to five times a day, miconazole mucoadhesive buccal tablets with 50 mg of active substance (FDA approved as Oravig) were considered more affordable systems due to their reduced systemic activity and good retention in oral mucosa [110].

Other treatments specific to systemic therapy, briefly presented in Table 2, are implied in the destructuration of the cell wall by the inhibition of β (1–3) glucan synthase in the case of echinocandins [111], or the alteration of protein synthesis and DNA damage in the case of 5-fluorocytosine. These drugs have poor local bioavailability and are proposed only for the intravenous route [107]. Echinocandins are an example of antifungals with lipopeptide structure and a high molecular weight that cannot pass the oral epithelium to ensure a local effect [107]. However, intelligent applications of echinocandins were recently discovered to promote antimicrobial action and avoid biofilm formation at the surface of biomedical materials. As the only echinocandin containing 2-ethylamine groups, caspofungin was used to functionalize aldehyde plasma polymer surfaces presenting reactive centers by irreversible binding through covalent bonds and exerting permanent antifungal activity [112].

Considering previous observations, the mechanisms of resistance are closely related to drug action and are mainly attributed to gene alterations for specific catalytic enzymes, which nullify the fungicidal effects, the use of efflux pumps, and the specific adaptive mechanisms of fungi cells, and not least, the generation of complex biofilms, which capture drug molecules and decrease therapeutic action [8,113].

The key factors to follow in the study of new antifungals for buccal delivery are related to the mechanisms of resistance and fungi cell dynamics, the local activity of the oral epithelium, and the knowledge of substances that can exert mechanistic strategies to efficiently inhibit fungi development and sustain drug passage through the tissue. Transcellular and paracellular routes, in a simple passive diffusion fashion, are the two pathways recognized for drug access at the level of the oral epithelium as a function of drug molecular weight, lipophilicity, electrical charge, pH, and mucoadhesion dynamics [62]. From a formulation point of view, the research of new pharmaceutical systems to target the oral mucosa is oriented toward the use of biocompatible polymers with bioadhesive properties [114], active penetration enhancers, enzyme inhibitors, and the development of nano-sized drug delivery systems [115].

Due to the high prevalence of *C. albicans* strains at the level of the oral mucosa, and considering the background focused on the mechanisms of pathogenesis and fungi behavior at the epithelial level, this study of the literature aimed to discuss possibilities and new trends through antifungal therapy for the buccal route. A large number of studies explored the antifungal activity of new agents or synergic components that may enhance the effect of classic drugs. For our purposes, it was of major significance to find a strong relationship between smart biomaterials or new compounds and their activity to find molecular responses and mechanisms that can conquer the multidrug resistance of fungi strains and transpose them into a molecular map.

There is no doubt that nanotechnology dominates this research field by repurposing new therapeutic agents from a broad area, specifically the nanocolloids domain, nanoparticle synthesis, and new composites that can be processed as biocompatible, tissue-friendly supports that exhibit superior antifungal activity. Furthermore, polymeric-based systems were found to join this group as new architectural building blocks of new drug delivery systems (DDS), satisfying good adhesion with the tissue, a modified release of the therapeutic agent, and sustained antifungal activity, with intimate mechanisms capable of destroying fungi structure. In another light, interesting reports complete a molecular map of antifungal mechanisms, with the introduction of nanoparticulate and polymer-based formulations as potential therapeutic solutions for oral candidiasis.

## 4. Biomaterials and Nanotechnological Approaches

Biomaterials are designed as biocompatible systems with therapeutic or diagnostic purposes. Tailored to be versatile, these structures are personalized to accomplish various requirements in the biomedical field, including pharmaceutical design. Implantable devices, regenerative scaffolds, and new drug delivery systems based on the use of new natural therapeutic molecules and synthetic compounds, or, more excitingly, traditional compounds modified or appropriately prepared as formulations tailored at the nanoscale level are a couple of actual directions in the development of smart biomaterials. Following the preparation process of a biomaterial and its physicochemical characterization, a second definitory step complies with the interaction with the host immune system, and the molecular signaling that guides therapeutic activity, but also cytotoxicity [121,122]. 

Particle size, shape, and surface characteristics are the main variables that guide the cellular dynamics of a biomaterial and are extensively researched in the case of nanocolloids, in both liquid-based formulations or solid states such as nanoparticles [122]. The large surface area of the nano-scale particles favors cellular passage through cell pores. Furthermore, the use of synthetic or natural biocompatible polymers such as polycaprolactone, polylactic acid, collagen, silk fibroin, alginates, and chitosan in the development of complex nanostructures is recognized as a practical approach to creating soft and safe systems [122,123].

The cellular responses are generated by the interactions of the newly synthesized formulation with the extracellular matrix, cell membrane, organelles, or nuclear sites and are oriented through molecular effects that mediate cell adhesion, cell migration, cell proliferation, the inflammation response, antimicrobial activity, cell growth and regeneration, and the reuptake of cellular nutrients [124,125]. With specific attention paid to *C. albicans* development on living or abiotic surfaces, Coad, B. et al. described the essence of designing antifungal-coated surfaces to avoid biofilm generation [126]. They emphasized the role of quaternary ammonium derivates as cationic functionalization agents on different surfaces. In a more advanced manner, the possibility to use antifungal agents as binding elements via covalent and non-covalent bonds, capable of disrupting the fungi cell wall, was revealed, as in the case of echinocandins [126,127]. Poly(methyl methacrylate) denture resin grafted with poly(1-vinyl pyrrolidone) was functionalized with miconazole via hydrogen bonding and hydrophobic interactions and a blockage was determined in the adhesion process of *C. albicans* and, by default, a diminished susceptibility to counteract denture stomatitis [128]. Nanoparticles are one of the most attractive systems whose definitory characteristics directly influence the penetration of fungi biofilms. In the same direction, tunable nanocolloids such as micro-/nanoemulsions serve as attractive soft pharmaceutical models able to target fungi cells and sustain antifungal activity due to the dispersion of the drug to the nanometric level [127]. Several models of biomaterials are further exemplified in this section, beginning with knowledge of nanocolloids and critical aspects concerning antifungal molecular pathways.

### 4.1. Nanocolloids for Old Molecules, but with Enhanced Antifungal Activity

#### 4.1.1. Microemulsions

Microemulsions are known as thermodynamically stable nanodispersions, commonly prepared as transparent mixtures composed of oil, water, a surfactant, and a cosurfactant using spontaneous emulsification. Surfactants and cosurfactants are two key elements implied in the stabilization process of the microemulsions due to a critical reduction in the interfacial tension and an increase in the surface area of the internal drops [129,130]. These thermodynamic effects favor the generation of various microstructures and the encapsulation of drug particles into the lipophilic or hydrophilic phase, resulting in liquid drops with hydrodynamic diameters up to 100 nm. In this way, the drug solubilization process can be satisfied, ensuring accessible pathways through biological membranes [131,132]. 

Particular knowledge was gained for the mechanistic pathways of microemulsions in the buccal delivery of antifungals to assess local alleviation. Al-Adham, J.S.I. et al. described several molecular actions of microemulsions as direct actives implied in the target of the cytoplasmic membrane of the microorganisms [10]. The alterations in the cytoplasmic membrane quantified by the release of intracellular K⁺ suggest a cascade of new events which culminates in cellular death. An oil-in-water system composed of Tween 80 17.3%, n-pentanol 8.5%, isopropyl myristate 5%, and water 69.2% was able to promote changes in the cell wall structure, favoring passage through the fungi cytoplasmic membrane and the damage of the cellular metabolism. A fungicidal effect on 90% of *C. albicans* cells was obtained after 110 s [10]. The disruption effect of microemulsions at the level of tight junctions was attributed to the presence of the stabilizers, which are known to be implied in cell membrane fluidization. This effect may justify the activity of similar microemulsions designed with classic azoles or natural fatty acids. To define a complex system and potentiate the effect of the imidazole-based compound clotrimazole, Kaewbanjong, J. et al. studied the antifungal activity of fluid- and gel-based microemulsions formulated with Tween 80 and Span 80 (1:1) 50%, isopropanol 20% and isopropyl palmitate 30%. The ex vivo release studies using chorioallantoic membranes that could mimic the oral mucosa emphasized the fluid microemulsions as immediate release systems and favored a large area of *C. albicans* inhibition. The addition of fumed silica 7.5% to microemulsions determined a controlled release of clotrimazole but an easily narrowed inhibition zone [133]. Furthermore, nanofiber-based scaffolds were previously designed as smart biomaterials with mucoadhesive properties and an effective and faster antifungal killing mechanism, which could conquer classic clotrimazole lozenges. Clotrimazole solubility can be improved via microemulsion design, viewed as an ingenious pathway that creates a possibility to incorporate lipophilic compounds and increase the antifungal activity and local retention at the minimum inhibitory concentration in the oral mucosa. A polymeric mixture composed of a chitosan–EDTA aqueous solution of 2% and polyvinyl alcohol (PVA) solution of 10% was combined with clotrimazole-based microemulsions prepared with oleic acid, Tween 80, and three types of cosurfactants, and underwent electrospinning. Three types of microemulsion-based nanofibers were studied [134]. The use of nanofibers obtained via the electrospinning technique is advantageous for oromucosal applications due to their extended surface area, high porosity, and reduced pore size, oriented toward both mucoadhesive traits and drug targets [135]. 

In the study of Monton, C. et al., natural approaches were discovered by accessing microemulsions with Tween 80 and PEG 400 as spray-based vehicles to entrap clove oil 2%. Rich in eugenol, clove oil was considered a natural alternative to antibiotherapy to avoid drug resistance and promote high antimycotic activity. Complementarily, PEG 400 was considered essential to assure an increased release of eugenol from microemulsion-based spray [136]. Eugenol, as a monoterpene phenol, is recognized for its antifungal activity related to the binding and inhibition of ergosterol biosynthesis. That is why clove oil was demonstrated to inhibit biofilm growth on various surfaces involved in biomaterial formation for implantable devices or food protection [137]. Considering eugenol’s structure, Ahmad, A. et al. studied eugenol’s antifungal activity on *C. albicans*, by repurposing semi-synthetic derivates that can interact with fluconazole. Eugenol-tosylate was the starting structure that was grafted with -CH_3_, -Br, -NO_2_, -Cl, and -CH_2_CH_3_ radicals and exhibited inhibitory effects on the lanosterol 14α-demethylase enzyme. The compounds act at MIC values of 1–62 μg/mL, under the value of 500 μg/mL specific for eugenol [138]. 

On the other hand, Tubtimsri, S. et al. proposed microemulsions as platforms for monolaurin solubilization in a mixture of Imwitor 742 14.38–18.22% and Kolliphor EL 41.23–47.94%. Monolaurin is a natural fatty acid, glyceride ester derivate of lauric acid that may exert antifungal activity in oropharyngeal candidiasis at an MIC of 0.09 μg/mL. To assess a better residence of the compound at the site of administration, the use of gel-based systems was considered a good choice, which was quantified by a release of monolaurin of up to 75% in the first hour. For its part, the fatty acid determined a large inhibition area of 43.7 ± 3.4 mm, as well as miconazole, but with an extended lag time of 2 h. Consequently, the free-drug microemulsions exhibited antifungal activity determined by the action of the surfactants at the level of fungi cell structures [139].

**Figure 3:**
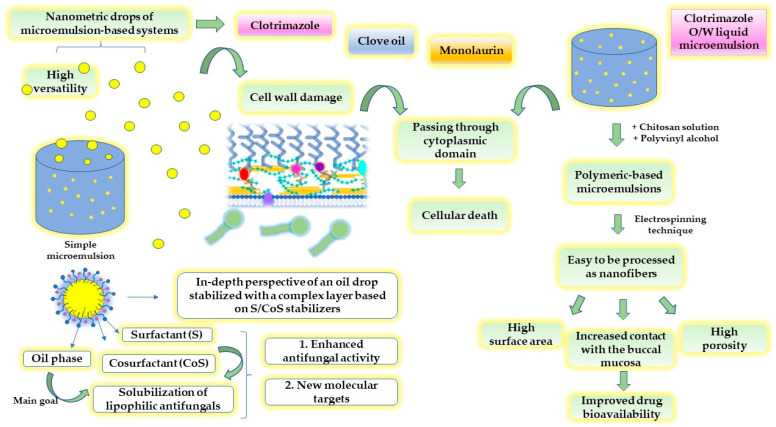
Microemulsion mechanism at the contact with the fungi cell wall and implications in drug processing and delivery.

Considering the previous examples describing the potential of microemulsions to solubilize and entrap antifungal drugs, Figure 3 presents an intuitive scheme with the main characteristics of microemulsions, their capacity to exert antifungal activity by targeting the cell wall of *C. albicans*, and the possibility to be processed as potential scaffolds for oral applications, as exemplified in the case of clotrimazole-based microemulsions gels processed as nanofibers.

#### 4.1.2. Nanoemulsions

Nanoemulsions are accepted as biocompatible systems, designed as an alternative to microemulsions, with a reduced concentration of surfactants but a high susceptibility to instability phenomena, which appear during the storage period. In this way, the reduced amount of the stabilizer enables the formation of droplets with an extended domain up to 200–500 nm. For this reason, high-energy methods are required to optimize the preparation process to obtain tiny droplets [140,141]. These systems are relatively new and have been extensively studied as promising candidates for the oral delivery of drugs [142,143], as well as skin delivery [144,145], but have rarely been used to create topical vehicles for the oromucosal route. Some worthwhile systems define an open window in terms of the undiscovered potential of nanoemulsions, beginning from the old nanoemulsion nystatin [146]. 

In the study of Fernández-Campos, F. et al., nystatin was examined as part of a nanoemulsion to assess new data concerning antifungal activity and tolerability by the ultrastructural analysis of oral tissue. Labrafac lipophile 25% as the oil phase, Labrasol 35.42% and Plurol Oleique 14.16% as surfactants, and Transcutol P 15.42% and Propylene glycol 10% as cosurfactants formed a suitable nanoemulsion vehicle that may act to aid nystatin solubilization and dispersion up to 138 nm, and then penetration through the mucosal tissue. A sprayable fluid-based system can assure an intimate contact with the mucosa, favoring pronounced antifungal action with an MIC of 0.78 μg/mL on *C. albicans* strains versus 1.56 μg/mL for the free drug [108].

In the study of Soriano-Ruiz, J.L. et al., clotrimazole was delivered in a nanoemulsion system prepared more simply than in the anterior case. Thus, Labrafac lipophile 10% was combined with a mixture 4:1 of two stabilizers, namely Labrasol and Capryol 90, in a total amount of 60%. a Lastly, propylene glycol 30% was added as a cosurfactant. It was emphasized that the nanostructured state of the clotrimazole-loaded nanoemulsion exhibits a better antifungal activity, due to a larger surface area of droplets, which creates an extended attachment on fungi cells. Furthermore, it was hypothesized that zeta potential is not only a stability parameter for the formulation process. The low charge values of the drops at pH~6 may determine interactions with the negatively charged phospholipids of fungi cells [147]. Hosny, K.M. et al. proposed complex self-nanoemulsion systems for oral candidiasis by using hydrogels as mucoadhesive supports and as hydrophilic dispersion mediums for various mixtures of clove oil 10–25%, Labrasol 45–70% and propylene glycol 10–30%. The use of hyaluronic acid as a biocompatible polymer, along with miconazole as an active molecule, was thought to determine multiple activities at the cellular level, oriented through tissue regeneration and an enhanced antimycotic activity, which was quantified by high permeation flux for the optimal formulation [148]. In another light, a recent study revealed the potential of oregano oil as a natural option to treat oral infections with respect to self-nanoemulsifying types of dispersions. Following the antifungal activity evaluation, it was observed that the inhibition area was correlated with the increase in oil and surfactant content. Maximum antifungal activity was predicted when oregano oil was settled at 18% and the surfactant mixture of Pluracare and Lauroglyocl FCC (1.25:1) was settled at 36% [149]. Thymol and carvacrol are phenolic components of the essential oil, responsible for enzymatic degradation in the fungi structure. Carvacrol is recognized to decrease SAP gene expression even in the fluconazole-resistant strains [150,151]. 

Flavonoids are other natural compounds that have been studied for antifungal activity. It was proved that kaempferol and quercetin exhibit antifungal activity against *C. parapsilosis* and *C. metapsilosis* cultures following several pathways, notably the dysregulation of nucleic acid synthesis and the inhibition of cellular adhesion, affecting the development of the biofilms [152]. Molecular docking studies showed that quercetin had an increased affinity to bind 14α-demethylase and nucleoside phosphokinase enzymes, acting on a similar mechanism of azoles [153]. To complete the quercetin profile, Lotfi, M. et al. studied its molecular effects after inclusion into a nanoemulsion vehicle. The treatment was studied on animal models to combat oral mucositis induced by 5-fluorouracil [154]. Oral mucositis is manifested as an inflammatory pathology that determines candidiasis development as a serious complication [155]. The active molecule has poor water solubility, and its solubilization was proposed to be conducted in a mixture of glyceryl monooleate as the oil phase, Kolliphor RH40 as a surfactant, and PEG 400 as cosurfactant, in a ratio of 1:8:1. Designed as a self-nanoemulsion template, its dispersion in water in a ratio of 1:5 determined the generation of an O/W quercetin nanoemulsion. The treatment with quercetin-loaded nanoemulsion was found to suppress the expression of Hif12 and NfkB inflammatory genes after 5-fluorouracil exposure [154]. It can be presumed that the inhibition of inflammatory events in chemotherapy-induced oral mucositis can counteract mycotic complications in the oral cavity and improve oral health. 

#### 4.1.3. Nanosuspensions

The particle diameter is considered the main parameter that will influence the therapeutic activity in the case of a pharmaceutical suspension. In this case, the nanosizing process offers important attributes in terms of stability and drug delivery. Firstly, the reduced particle size at the nanometric level opposed the outbreak of drug sedimentation by decreasing the sedimentation rate, followed by the high accessibility of nanoparticles into the area of administration. The application of nanotechnology in the research area of suspensions offers significant advantages for various routes of administration, such as oral, topical, buccal, nasal, or ocular, to solve bioavailability challenges [156]. To assess antifungal activity, nystatin nanosuspensions were prepared using commercial suspensions with nystatin of 100,000 IU/mL. The process consisted of the water dilution of the initial product and wet milling to attain nanometric particles up to 800 nm, as a function of nystatin concentration. For adequate nanosuspensions with nystatin 40,000 IU/mL, particle diameter varied between 96 and 237 nm, while for a concentration of 50,000 IU/mL, the diameter was between 75 and 365 nm. Over the course of in vitro studies in mice, the inhibition effect of the nanosized nystatin on *C. albicans* development occurred in 24 h. The rapid onset of nystatin activity is favorable for the use of a nanosized formula in oral candidiasis [157]. Modern approaches have updated the current requirements, taking into consideration the increase in the biocompatibility of a product by accessing mucoadhesive systems based on polymers. Hence, nanosuspensions can be tailored as sophisticated systems by appealing to polymeric-based vehicles, which can be processed as advanced structures for local delivery, such as films. This purpose was considered for clotrimazole nanosuspensions stabilized with O-succinyl chitosan, which was blended with a polymeric mixture composed of catechol-functionalized hyaluronic acid and polyvinyl alcohol. The polymeric matrix behaves as a strong connector with mucus glycoproteins [158] and determines powerful interactions which assure the increased action of clotrimazole. On the other hand, polymers act in the same manner as protective agents for living cells to avoid the toxicity of high concentrations of clotrimazole. The nanosuspension had an MIC of 12.5 μg/mL and resulted in a higher area of inhibition of 9.33 ± 0.58 cm, which was correlated with the ability of the nanosuspension vehicle to solubilize drug molecules [159]. 

#### 4.1.4. Nanostructured Lipid Carriers (NSLc)

Nanostructured lipid carriers are the last representatives of the nanocolloids class chosen to serve as reliable models for the antifungal activity enhancement of the old drug molecules. These systems are lipid-based variants of nanoparticles, prepared with solid lipids and surfactants, which act as good solubilization agents and carriers for lipophilic drugs. For buccal delivery, nanostructured lipid carriers were designed for the incorporation of fluconazole or miconazole. In the first case, 17 NSLc with fluconazole 0.03–0.07 g were prepared using glycerol monostearate 1.5–2.5 g as the lipophilic phase, Tween 80 1–3 g as a surfactant, and distilled water up to 100 g. The hot homogenization technique was applied to form solid lipid nanoparticle formulations. The systems were optimized with consideration of physical parameters, and the antifungal activity was studied on five optimized systems formulated with Tween 80 2–3%. It was found that Tween 80 promotes a large surface area for lipidic nanoparticles and assures their stabilization. Furthermore, the interaction of the drug with the lipidic domain of nanoparticles influenced antifungal activity. If the drug molecule is highly partitioned in the lipidic content, the release rate into the fungi strain will decrease, and the time to assess a therapeutic effect will be extended. On the other hand, it was presumed that the hydrophobic surface of NSLc had an affinity for fungi cells, determining a localized effect [160]. The attention paid to formulation parameters is directly linked with therapeutic potential and the intimate mechanisms that target fungi destabilization. 

Mendes, A.I. et al. chose to encapsulate miconazole into NSLc based on Gellucire 43/01—7%, Mygliol 812—3%, Tween 80—2%, and benzalkonium chloride 0.5%, and obtained systems with particle diameters of 200 nm and sustained antifungal activity in reduced doses. Thus, the initial miconazole dose of NSLc of 0.3% was reduced to 0.12% after imposing the hydrogelling technique and embedding NSLc in a hydrogel prepared with Gelling PFC 1%, glycerin 3%, and benzalkonium chloride 0.01%. The antifungal activity of the prepared hydrogel was expressed as a zone of inhibition of 25.5 mm, similar to that of the commercial product, 24.3 mm [161]. The inclusion of NSLc in a hydrogel network determines a close contact with the mucosa, a better adhesion, and a sustained long-term release of the active molecule by surpassing potential adverse reactions.

The optimization study of Hosny, K.M. et al. included sesame oil as a second lipophilic agent capable of potentiating miconazole activity as part of an NSLc. One of the strengths of this research included the use of Labrasol surfactant solution, which became a factor in a nanosized dimension of the NSLc and promoted proper drug permeation into the oral tissue. According to the evaluation of the ulcer index on rats treated with *C. albicans* colonies, the synergism between miconazole and sesame oil in alleviating mucosal ulcerations was proved. This fact can be justified by the antioxidant activity of the natural oil, associated with the molecular action of miconazole [162].

### 4.2. Inorganic and Organic Nanoparticles

Nanoparticles (NPs) are a group of agents designed to exhibit targeted action against fungi development in all phases of growing, including tolerant biofilms. Several significant research works were dedicated to specific synthesis methods to obtain biocompatible inorganic agents in the nanoparticulate form [127]. Although a high degree of interest has been given to the study of silver, copper, zinc, or selenium-based nanoparticles [127,163], complex approaches were intended to tailor specific agents such as polymeric nanoparticles [164], polymer-based inorganic nanoparticles [165], magnetic nanoparticles, functionalized derivates [166] and peptide-based nanoparticles [167]. The attention paid to the conjugation capacity, particle dimension, surface charge, and cell–material interactions of surface-sensitive nanoparticles opened a broad perspective in terms of antifungal drug delivery. In this regard, the possibilities to synthesize and analyze potential agents have lasting interest, revealing new mechanisms that can conquer the activity of classic drugs.

The conjugation of nystatin and fluconazole with silver via the borohydride reduction of AgNO_3_ determined the generation of nanoparticles with superior antifungal activity. An inhibition of 50% was obtained for the nystatin nanoconjugate and 62% for the fluconazole one, at an MIC value of 300 μg/mL. Thus, it exceeded the initial inhibitory effect of 30–32%, specific for the free drugs. The mechanisms by which nanoconjugates affect the cellular level of *C. albicans* were suggested to be related to pore formation, in the case of nystatin silver-based NPs, continued with general cellular destructuration under the effect of fluconazole silver-based NPs. Additionally, silver in its nanoparticulate form is recognized for the production of reactive oxygen species and attachment to sulfur-grafted proteins. These effects directly attack fungi cells by inducing alterations and intracellular leakage [168]. To complete these statements, another study that regarded silver action but at the microscale level highlighted the influence of αAgVO_3_ microcrystals on *C. albicans*. The oxidative stress produced under the Ag action determined fungistatic and fungicidal effects [169].

Silver NPs are prepared using techniques that assume chemical, photochemical or electrochemical reduction, laser ablation, and lithography. These methods are time-consuming, laborious, and present some disadvantages in the chemical synthesis of pharmaceutically acceptable agents, such as the use of a high number of chemical reagents, obtaining uneven nanoparticles, reduced reaction yield, and environmental drawbacks [170]. Bioinspired alternatives have been selected to obtain biocompatible nanoparticles in a sustainable manner [12,171]. In the study of Zangeneh, M.M. et al., simple silver nanoparticles were synthesized using an aqueous extract of *Allium saralicum* sp. leaves [12]. The plant has an oriental origin, being appreciated for its antioxidant and antimicrobial activity due to the high content of linoleic acid methyl ester [172]. Hence, the synthesis process was based on the reduction of Ag^+^ from AgNO_3_ to elemental Ag^0^ in the presence of polyphenols as part of the 2% (*w/v*) leaves extract. The reaction process ended with the precipitation of silver NPs, and the reaction product was analyzed. Both polyphenols and silver NPs were identified by applying UV–VIS spectroscopy, being defined as polyphenol-specific bands for π→π* transitions at 235 and 305 nm. A silver-specific band was detected at 435 nm. Over the antifungal evaluation of *C. albicans* strains, an MIC value of 62 μg/mL was obtained. *C. krusei* and *C. guillermondi* were found to be more sensitive to silver NPs, being determined to have MIC values of 31 μg/mL [12]. It can be appreciated that green synthesis based on plant extracts represents a non-toxic, eco-friendly approach to generating nanoparticles with special antimicrobial activity [170]. Many attempts have been described in the literature to support this direction. Plant extracts obtained from *Acorus calamus*, *Aloe vera*, *Brassica rapa*, *Eucalyptus hybrid*, *Moringa oleifera*, *Nelumbo nucifera*, and *Vitex negundo* are a few examples of reaction substrates rich in polyphenols that may guide the synthesis of silver NPs [12]. In the study of Abdallah, B.M. et al., silver-based NPs were synthesized in the presence of a leaf extract derived from *Lotus lalambensis* Schweinf and explored for their antifungal potential in oral candidiasis. The research aimed to present the synergism between silver NPs and the leaf extract containing 5′-hydroxy auraptene as an inhibitor of *C. albicans* development. The synthesized nanoparticles of 6–26 nm resulted in a larger area of inhibition of 19 mm at MIC values of 50 μg/mL, which was superior to the plant extract in the same concentration. At an MIC of 100 μg/mL, the fungal growth was suppressed. In combination with the plant extract, silver NPs determined the largest inhibition area of 24 mm at a concentration of 50/100 μg/mL, explained by the suppression of fungi growth, adhesion, and biofilm formation. The associated molecular events sustained a decrease in *C. albicans* oxidative enzymes and intracellular glucose and trehalose release. Finally, the ultrastructural analysis using microscopic techniques outlined an invasive effect of the extract–nanoparticle combination, determining pore formation, intracellular content loss, and cell death [173]. In-depth attempts at elucidating the molecular action of silver NPs were described in the study of Mare, A.D. et al. The same synthesis principle was maintained by using the beech bark extracted from *Fagus sylvatica* L. Nanoparticles synthesis was performed following two pathways that consisted of using two solutions of 1mM, namely AgNO_3_ and CH_3_-COOAg solutions. It was revealed that the nanoparticulate products prepared with the acetate solution caused the downregulation of SAP2 expression, which is known to be involved in host cell invasion [174]. To further complete the mechanistic map of silver NPs, Jia D. and Sun W. focused their attention on the synergistical effect of the nanoparticles and fluconazole to suppress *C. albicans* activity. The central aspect of the study was proposed to be the manner in which silver NPs can counteract fluconazole-mediated resistance, with double significance in terms of both the topical and systemic activity of azoles. Besides the reduced MIC values of AgNPs of 32 μg/mL, compared to the value of 128 μg/mL of fluconazole, the use of the agents in *C. albicans* isolates caused fungi inhibition only in the early stages of biofilm consolidation up to 6 h, without exerting activity in the maturation stages. The associated agents increased the length of action time up to 12 h, which may define the importance of a proper antifungal therapy initiation as soon as possible in the early stages of diagnosis. Furthermore, it was found that a concentration of 30 μg/mL AgNPs sustained ergosterol depletion by inhibiting the expression of ERG1, ERG11 and ERG25 genes. This effect determines a fluidization of the membrane, affecting the activity of the Cdrp1 and Cdrp2 efflux pumps, responsible for azoles resistance. The in vivo studies on mice were favorable for the use of AgNPs to increase survival rate, without inducing toxicity [13].

Copper oxide NPs are an example of potential antimicrobial agents that can be prepared to create complex nanostructures by using biocompatible and biodegradable polymers. In a recent study, CuO NPs were combined with poly-ε-caprolactone and then electrospinned, resulting in nanofibers with diameters in the range of 925–1080 nm. CuO NPs and their nanofibers were physically described and tested on *Candida* species with development into the oral cavity, including *C. albicans*. The exposure of *C. albicans* to CuONPs-based polymeric nanofibers at a concentration of 200 mM caused alterations in mycelia integrity, remarked as deformations, distortions, and contractions. CuO NPs have a major effect on fungi destructuration, while the polymer creates a biocompatible platform that sustains contact with the oral mucosa and the release of NPs [175]. Similarly, TiO_2_ and ZnO NPs were studied for their antifungal activity as potential alternatives to amphotericin, but their effect was favorable at higher MIC values than the antifungal MIC [176]. 

**Figure 4:**
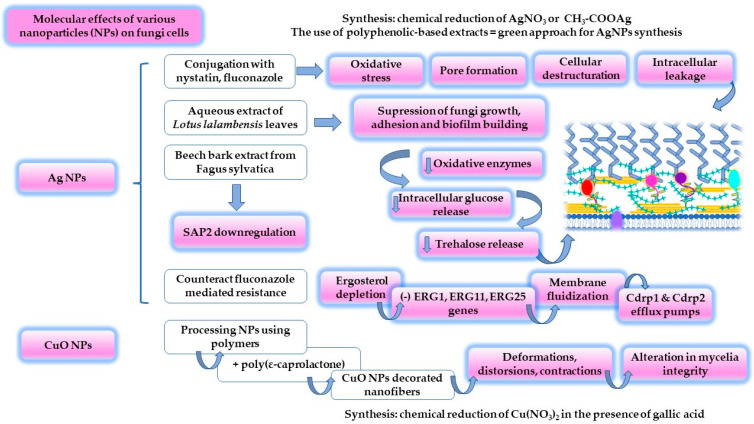
Antifungal activity and molecular effects of silver and copper oxide inorganic nanoparticles in *C. albicans* strains.

Based on the knowledge assessed in the studies mentioned above, a molecular map was composed, and is presented in Figure 4. The antifungal activity and molecular effects of the most valuable systems designed using silver and copper nanoparticles are presented herein.

Magnetic NPs of ~4 nm, obtained using the coprecipitation of two iron salts, were studied for their antifungal activity as part of a liquid crystalline system formed with Brij O10 as a surfactant, isopropyl myristate as the oil phase, and distilled water. The complexity of the colloidal system was defined by the use of an ethanolic propolis extract with antimicrobial properties. The formulation was proposed as a delivery system with prolonged release in the treatment of periodontal disease, which was previously described as a predisposing factor for candidiasis development. The fungicidal activity of the system was increased when an alternative external magnetic field was applied [166].

As a final model with valuable older notes that can be regarded in the study of new nanoparticulate formulations for buccal delivery, organic nanoparticles of poly(ethyl cyanoacrylate) were the central subject of antifungal activity assessments. Looked at from a different perspective and compared with anterior examples, polymeric-based nanoparticles were considered appropriate therapeutic agents without being only an entrapment resource. The emulsion polymerization technique was applied, and the antifungal polymeric NPs were generated under the stabilization effect of cationic, anionic, and non-ionic surfactants. Thus, the positively charged NPs characterized by high zeta potentials of +31.3 mV (for cetrimide) and +26.2 mV (for cetylpyridinium chloride) represented the main potential agents for antifungal activity [164].

### 4.3. Graphene-Oxide-Based Biomaterials

Graphene was first isolated in 2004 by Andre Geim and Kostya Novoselov. They have drawn attention to the mechanical exfoliation technique of graphite to obtain single sheets of graphene. The new method inspired the research world and was awarded the Nobel Prize in Physics in 2010, paving the way to exciting discoveries in material sciences [177]. Graphene is described as a single-layer two-dimensional (2D) carbon-based material with a honeycomb appearance, characterized by stable sp^2^ bonds [178]. Highly researched for its characteristics based on good mechanical strength, high surface area [179], optical transparency, elasticity, and electronic properties [180], graphene constitutes an interesting bidimensional structure that can be functionalized to serve in tissue engineering [181], implants [182] and biosensor fabrication [183], and the design of biomaterials as drug delivery supports, due to its biocompatibility with human living tissues [184]. One of the most affordable chemically active derivates of graphene is represented by its oxidized form, which is usually obtained using the Hummers method, in the presence of oxidizing agents such as KMnO_4_ in a concentrated medium of H_2_SO_4_, under a rigorous step-by-step temperature control [185,186,187]. Subsequently, graphene oxide can be converted into reduced graphene oxide via chemical or thermal reduction. Graphene oxide (GO) possesses multiple available groups with a covalent character, such as epoxydic, hydroxylic, and carboxylic moieties, which are ready to be functionalized with active compounds, polymers, and inorganic nanoparticles to create attractive nanocomposites for drug delivery [188]. In the same manner, its hydrophilicity assures the generation of water-based colloidal dispersions as a background for pharmaceutical applications [189]. Graphene-based biomaterials have multiple applications in dentistry, where materials for dental recoveries, such as new graphene-based acrylic resins [190] or adhesive sialinized GO-based NPs for dental brackets, can be good alternatives to improve oral health by ameliorating the imbalances in the oral microbiota, improving biofilm templating and reducing the risk of cariogenic events [191].

Likewise, novel platforms for the delivery of antimicrobials and regenerative agents are of major significance to conducting medicine at a safer, high-quality, and modern level [192]. The antimicrobial activity of graphene derivates is addressed at the same time to combat oral fungal infections [193]. It is essential to highlight that the antimicrobial mechanisms are closely related to the mechanical properties of the 2D structures and nanostructures based on graphene. Graphene derivates in a 2D state induce mechanical stress and prevent microbial cell development by nutritional depletion. In addition, the nanoparticulate structures initiate microbial cell penetration and destructuration. Lastly, to target nuclear sites and damage DNA, oxidative stress was considered a specific mechanism for the oxidized form of the 2D GO-based layers, being more pronounced than in the case of nanoparticles. Proportionally, cytotoxic events must be regarded as a function of the number of the oxygen-based functional moieties grafted in the GO structure [190]. In this case, GO functionalization with polymers and surfactants represents an ingenious approach to decrease toxicity and attain improved therapeutic activity [188].

Graphene oxide nanocomposites are versatile candidates to sustain local antifungal activity for gynecological purposes, wound regeneration, and the targeting of specific infections in mucocutaneous tissues. In this regard, Li, C. et al. studied nanocomposites prepared with graphene oxide and silver nanoparticles as carbon nanoscrolls. Elongated structures were obtained from graphene oxide sheets filled with silver NPs by applying sonication. The antifungal activity was studied for GO, GO-based silver NPs and the nanoscrolls. It was observed that GO-based nanoscrolls exhibited superior antifungal activity at an MIC of 0.125 μg/mL, compared with the simple GO-based silver NPs which achieved doubled MIC values of 0.5 μg/mL. At the same time, GO itself did not induce any action on fungi strains. SEM images proved the lysis process of *C. albicans* and *C. tropicalis* fungi cells, and the loss of intracellular content after prolonged contact with the nanoscrolls. GO-based nanoscrolls were considered reliable models of the slow release of silver NPs, thus avoiding the undesirable cellular toxicity produced by silver accumulation [194]. In a similar direction, Cui, J. et al. emphasized the antifungal properties of GO silver-based NPs and their cellular safety. GO structures were obtained using the hydrothermal method and polyvinyl pyrrolidone (PVP) was selected to stabilize GO sheets. In the presence of 1 mM AgNO_3_, Ag NPs of 12 nm were formed with a uniform distribution in the GO structure. The antifungal activity was quantified as the complete inhibition of fungi growth at the same concentration of 50 μg/mL as in the case of GO-based Ag NPs, and the diameter of inhibition was 21 ± 4 mm. Furthermore, the inclusion of Ag NPs into the GO structure increased cell viability and reduced apoptotic and necrotic events, as was proved in experiments on HacaT cells [187].

When polymers enrich graphene oxide, new valuable physiologically acceptable composites are created to combat the dissemination of fungal infections. Cheong, Y.-K. et al. used a chemical conjugation reaction for amide bond formation to produce PEGylated graphene oxide (PEG-GO) and PEGylated expanded graphene oxide (PEG-EGO). It was demonstrated that copper NPs are efficient against *C. albicans* due to pore formation on the yeast cell surface and apoptotic action. On the other hand, the PEG-GO composite has a superior antifungal effect compared with PEG-EGO, GO, and EGO, but under the activity of Cu NPs. This is why a synergism of PEG-GO and CuNPs promoted high adhesion on the yeast spores and hyphae, followed by their deformation. From a physical point of view, the antifungal effect of the PEG-GO derivate was correlated with the zeta potential value of −41.9 mV. In this regard, these structures have powerful aggregative stability, maintaining their properties over time. As a final result, a reduced *C. albicans* cell viability was obtained at a concentration of 500 μg/mL of PEG-GO and CuNPs in a ratio of 30:70 [195].

In another light, the entrapment of antifungals in the structure of GO can be a real opportunity to enhance antimycotic activity. Few but promising research works have studied azoles as active molecules for GO binding. The advantages of these formulations are described as attempts to assure high stability, a modified release of the active ingredient, and a synergism created with the aid of GO as a substrate of release [14,196]. Asadi Shahi, S. et al. developed safe GO structures by including fluconazole via a chemical method. The antifungal activity on *C. albicans* colonies was obtained at MIC values of GO/fluconazole of 400/9 μg/mL. The mechanism was proposed to act in multiple directions, implying fungi cell lysis and chemical oxidation via GO, followed by DNA damage. The GO/fluconazole system was found to interfere with the adhesion step of fungi cells, promoting gene downregulation [196]. 

The large surface area of GO represented a new beginning for the design of biocompatible GO-based oral films. Thus, Huang, J. et al. projected GO-based polymeric films for the local delivery of clotrimazole, using chitosan and alginate as proper mucoadhesive polymers that modulate the antifungal activity of clotrimazole via electrostatic interactions. There was a graphical explanation of the chemical interactions between ${-\mathrm{COO}}^{-}$ and ${\mathrm{NH}}_{3}^{+}$ groups of the two polymers, followed by supplementary interactions at the addition of clotrimazole and GO into the polymeric matrix. The inclusion of GO was advantageous when increasing clotrimazole release, and at the same time, expanded the area of *C. albicans* inhibition to 17.1 mm. This aspect was suggested to be sustained by the interactions between the polymers and GO, and then between GO and clotrimazole [14]. In their recent study, Huang, J. et al. proposed valorization of the polymeric mixture, including GO and clotrimazole in the form of polymeric foam with the same therapeutic purpose. It was observed that GO sustained the mobilization of clotrimazole from the polymeric matrix by increasing the release rate as a result of concealing the electrostatic interactions between alginate and the drug [197].

**Figure 5:**
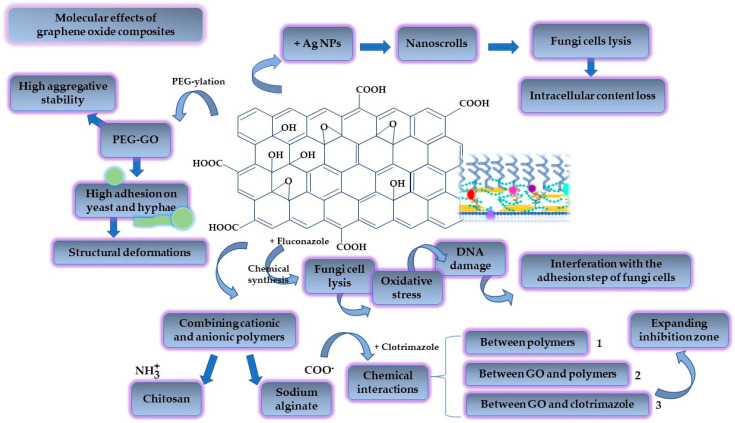
Antifungal activity and molecular effects of graphene oxide biomaterials in *C. albicans* strains.

To conclude, graphene oxide has a high potential for drug delivery and can be successfully integrated into novel antifungal platforms. The association between inorganic nanoparticles, antifungal drugs, and mucoadhesive polymers promotes synergistic actions and favors fungi cell death. In Figure 5, a summary of graphene oxide applications for the buccal route is described, emphasizing the main antifungal effects.

### 4.4. Polymers for Tailored DDS with Antifungal Activity

Mucoadhesive polymers are studied as functional biomaterials that can enhance the therapeutic activity of drugs in buccal delivery. By adequate processing, these structures assure the attachment of the pharmaceutical formulation to the mucosa without being removed through the salivary flow or enzymatic processes [64]. Polymers’ versatility is also recognized in their ability to sustain drug delivery after incorporation into nanocolloidal dispersions, nanoparticle supports, or complex nanocomposites, as previously discussed. The interest becomes greater when polymers have the power to interact with drug molecules or to be grafted with functional chemical groups, initiating the antifungal activity in this route [198].

Firstly, at the level of the oral mucosa, the salivary glands permanently ensure a proper level of saliva, which is based on the proportion of water being 99% and organic substances 1%. Saliva assures the troficity of the oral tissues and maintains adequate hydration and protection via immune defense. On the other hand, it participates in the initial biochemical reactions of the first phase of digestion. Thus, the presence of enzymes such as lipases, proline-based proteins, peroxidases, amylases, and glycoprotein-based mucins, as well as pH changes, influence the activity of a drug molecule [64,199,200]. The use of mucoadhesive polymers in the formulation of oromucosal systems is inspired by the protective and barrier-like behavior of the mucus. At the normal oral pH of 6.2–7.0, it forms a resistant pellicle that adheres to the epithelium [201,202]. The presence of sialic acid and glycoproteins determines the effect of anionic polyelectrolyte and, by default, the viscoelastic properties of the mucin. Under the influence of calcium efflux, the electrostatic repulsions of the negatively charged molecules determine the elongation of mucin chains. Furthermore, the non-covalent interactions between the mucin chains favor hydrogen bond formation, and electrostatic and hydrophobic interactions, which represent the chemical basis for a viscosity increase and the generation of a polymeric network at the point of contact with water molecules [203]. 

Consequently, mucoadhesion describes the capacity of a material to adhere at the level of the epithelium and interconnect with the mucosal film to sustain prolonged retention, but also a modification of cellular permeability [202]. This intimate contact can be found under the concept of bioadhesion [204], a phenomenon that can be explained using a series of theories. The fundamentals of polymer–mucosal film interactions represent a cumulus of electronic, adsorption, wettability, diffusion, breaking, and mechanical phenomena which serve to develop polymeric-based materials such as mucoadhesive tablets [110], oromucosal gels [205], polymeric films [14], or polymeric-based supports enriched with nanoparticles [175]; the last two cases define advanced drug delivery systems for antifungals delivery.

There is a need to develop new antifungal agents to avoid multidrug resistance to classic antibiotherapy. Thus, polymers are promising biomaterials that can be processed to exhibit antifungal activity. Recent pursuits proposed various manners in which polymers may be adapted to these requirements by the modification of the polymeric structure with specific moieties derived from antifungal drugs, the modification of polyenes and echinocandins, the synthesis of composites with the inclusion of inorganic or organic compounds, or designing polymers as inclusion agents for various drug molecules [198]. Groups of peptide-like polymers, inspired by the activity of antimicrobial peptides, have been suggested, with activity on resistant *C. albicans* and other fungi strains. Nylon-3-copolymers containing AMP structural groups were synthesized by the route of the anionic ring-opening polymerization of β-lactams and were codified as MM-TM, DM-TM, and NM. However, in-depth studies are required to assess valuable data concerning their antifungal activity because their action was considered limited to yeasts only [206,207]. In the study of Liu, R. et al., nylon-3-polymer with 20 β nm subunits was considered highly active on resistant *C. albicans*, exerting fungicidal activity at 3 μg/mL, with minimal toxicity on fibroblasts. Hemolytic activity and the discovery of derivates with selective toxicity on fungi cells represent a challenge to finally conclude that these polymers can be safely integrated into formulations with clinical efficacy [208]. Considering these aspects and the broad array of antifungal polymers, we chose to exemplify polymeric-based agents with antifungal activity intended to have a predilection for buccal delivery, and their mechanisms of action.

Polymeric micelles are interesting complex nanovehicles for drug delivery, designed to solve the major drawbacks of lipophilic agents, specifically solubility, inadequate drug size, or drug passage through mucosal tissues [209]. Such a type of carrier was proposed to include itraconazole as a candidate for the transmucosal route via the oral mucosa. This insight can be considered ambitious, with the purpose to integrate a usually accepted systemic drug into a nano-based polymeric vehicle. In the study of Suksiriworapong, J. et al., polymeric micelles were formed using D-α-tocopheryl poly(ethylene glycol)-1000 succinate (TPGS). The polymer can establish covalent interactions with the cysteine groups from the mucus film by disulfide bonds. In this way, a passage opportunity is created for the drug to attain deeper epithelial layers and then the vascularized domain. Similarly, a coupling reaction between TPGS and L-cysteine was considered favorable for the pharmaceutical relevance, mucoadhesion, and antifungal activity of a polymeric complex of TPGS/TPGS-Cys mixed micelles of 8–10 nm. The inclusion of itraconazole into polymeric micelles oriented itraconazole deposition into the lipophilic tocopheryl core. In consequence, the system positively affected the decrease in MIC values by 1.35-fold, from 0.250 μg/mL to 0.185 μg/mL [210]. 

Zhou, L. et al. created nano-inspired micelles to load luteolin as a natural active compound for oral candidiasis. Their study proposed the creation of a tri-block copolymer satisfying two main actions, namely luteolin solubility enhancement, promoted by a diblock polymer of monomethoxy polyethylene glycol–poly-ε-caprolactone (MPEG-PEC), but also an inhibitory action on *C. albicans* by the inclusion of cationic polyethyleneimine (PEI) [211,212]. The advantages of the triblock copolymer were the biodegradable nature of the system, the reduced cytotoxicity due to the presence of PEG blocks, the inclusion of luteolin into the PEC hydrophobic core, and the overall antifungal mechanisms. Thereby, the positively charged nanosized micelles are concentrated in the fungi cells under electrostatic forces. Cationic moieties of micelles have an affinity for the anionic charges of the biofilm. Finally, their adhesion and permeation through the biofilm structure resulted in fungi cell damage. The antifungal effects were sustained by reduced MIC_50_ and MIC_80_ values in both planktonic and biofilm states of *C. albicans* [213]. 

New antifungal solutions for resistant oral candidiasis were proposed by Mady, O.Y. et al. They developed polymeric oral films based on carboxymethylcellulose and polyvinyl alcohol to support the antifungal synergism of a combination based on a miconazole base and urea. The synergism was quantified and accepted due to a miconazole MIC reduction from 32 to 0.0625 μg/mL, when combined with urea, followed by high inhibition diameters of 30–40 mm. Their activity led to mycotic cellular death and necrosis, with cytoplasm rupture and intracellular content release. In this case, mucoadhesive polymers sustained the adhesion and release of the antifungals [15].

Kraisit, P. et al. merged the film properties of chitosan with the inclusion of solid lipid nanoparticles as NSLc for fluconazole delivery at the oral level. The inclusion of fluconazole into NSLc prepared with glyceryl monostearate and stabilized with Tween 80 solved its solubility problems, while its release to the oral mucosa was hypothesized to occur due to the hydrophilic character of chitosan. The addition of NSLc into chitosan films can affect the mucoadhesive properties of the polymer. The bioadhesive effect was considered to be enhanced in high levels of Tween 80 and chitosan, which form hydrogen bond interactions with the mucus. Considering the antifungal activity, the nanosized level of NSLc of 63.73–265.8 nm may determine a facile penetration of the fungi cell membrane and attenuate antifungal drug resistance [214]. From another perspective and taking into consideration chitosan as a cationic polymer, its structure inspired the development of several cationic antifungal polymers. Chitosan synthesis implies the partial deacetylation of chitin, resulting in a linear compound based on D-glucosamine and N-acetyl-D-glucosamine units [215]. Chitosan possesses essential properties related to drug delivery, specifically mucoadhesion promotion and the increase in drug penetration. It has a biodegradable character, the ability to be integrated in complex polymeric drug delivery systems, hydrogels, and nanocolloids, and not least, it presents regenerative and antimicrobial qualities [216]. Although these properties seem to describe an ideal structure for biomedical applications, chitosan needs proper processing due to its hydrophilicity and limited solubility [198,216]. Thus, the modification of its structure determines the generation of chitosan derivates with N-substituted groups via alkylation. Few examples can be mentioned and are not limited to the N-nucleophilic substitution of chitosan with 4-chlorobutyril chloride, resulting in an ammonium derivate [217], or the proceeding reaction into a combination of Schiff bases, followed by reduction to form N,N,N-trimethyl chitosan [218]. These reactions were available to enhance the antifungal properties of chitosan in food and water processing. Researching new chitosan-derived antifungals with therapeutic purposes represents a challenge for future discoveries.

The polymer contribution to the antifungal activity on *C. albicans* biofilms was suggested by Al-Ani E. and Heaselgrave W. Hence, thymol was considered a powerful antifungal agent for oral candidiasis when combined with Poloxamer 407 as a polymeric surfactant. Subsequently, the polymeric carrier effect was compared with a second thymol formulation based on hydroxypropyl methylcellulose. Remarkable results were obtained when Poloxamer 407 was combined with thymol. The surface activity of the polymer determined the solubilization of the active compound and supplementary inhibitory effects on *C. albicans* biofilms were proved at higher concentrations of thymol and lower concentrations of Poloxamer 407. The inhibitory effect related to Poloxamer 407 activity was considered to be based on the suppression of the aggregative activity of the proteins of the biofilm [219]. Poloxamers represent triblock copolymers defined as polypropylene oxide–polyethylene oxide–polypropylene oxide structures with surfactant activity, applications in drug solubilization, and unique thermoresponsive gelling properties which recommend it for applications in buccal delivery [220]. Furthermore, their ability to modulate drug release into tissues following the prolonged-release pathway can be an essential clue to the preference for the inclusion of antifungals into poloxamer-based therapeutic templates [221,222].

**Figure 6:**
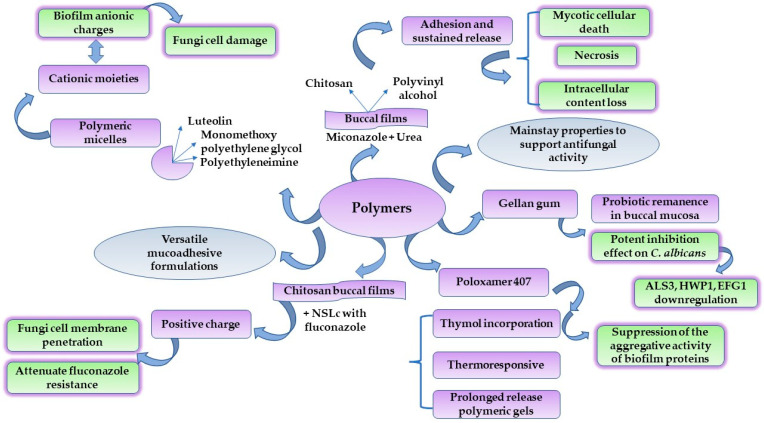
Antifungal activity support and molecular effects of polymer-based biomaterials.

Lastly, a natural alternative to support the antifungal activity in the oral mucosa was presented in the study of Ribeiro, F.C. et al. Their study was based on the assessment of the antifungal activity of probiotics, namely *Lactobacillus paracasei* 28.4. To protect and preserve probiotic activity, a biocompatible polysaccharide was proposed as an encapsulating agent. Gellan gum (1.0–0.6%) was considered appropriate to sustain the formulation process. The use of gellan gum 1% was favorable to permit probiotic remanence in the oral cavity for up to 10 days. Thereby, a study on murine models demonstrated the antifungal activity of polymeric-based probiotics. The inhibition of *C. albicans* activity in the oral mucosa was correlated with the probiotic effects upon the downregulation of several genes implied in biofilm architecture, namely ALS3, HWP1, EFG1, and CPH1 [223,224]. In Figure 6, valuable polymer applications for the buccal route were emphasized, with an emphasis on the antifungal activity obtained after testing new polymer-based biomaterials.

To support a therapeutic activity in the oral cavity and a sustained release, the selection of biocompatible polymers is of major significance, being generally discovered in the development of mucoadhesive nanocolloids, nanoparticle delivery, or the design of nanocomposites and mucoadhesive scaffolds. Polymers are versatile supports that enhance the antifungal properties of natural or synthetic agents, and have been documented as valuable biomaterials that may complete the mechanistic pathways of old and new drugs.

## 5. Conclusions

Oral candidiasis remains a complex pathology with a high power of dissemination through a systemic state. Mainly occurring in immunocompromised patients, mucocutaneous infections with *C. albicans* are recognized to be of high emergence and need an early diagnosis for a favorable prognosis. The development of oral candidiasis is based on multifactorial mechanisms concerning local tissue imbalances and poor local immune defense. These events culminate with real changes in the oral metabolism, mediated via gene regulation. The challenges of drug delivery research present a unique invitation to develop new strategies to overcome the multidrug resistance of fungi strains. Classic drugs cope with the most sophisticated mechanisms of resistance of fungi, which target catalytic enzymes and gene regulation. Biomaterials are promising carriers to enhance the antifungal activity of classic drugs by improving mucoadhesion in oral tissue. Nanocolloids are ideal formulations to solve the solubility problems of lipophilic drugs, exerting destructuration effects on fungi cells.

Furthermore, newly synthesized inorganic nanoparticles from vegetable resources are considered eco-friendly and easy-to-synthesize systems that can interfere with biofilm development and gene regulation. Subsequently, organic composites based on graphene oxide are demanded as versatile platforms that may covalently interact with drug molecules and polymers. It was pointed out that graphene-oxide-based materials such as silver nanoparticle-decorated graphene oxide have superior antifungal activity, protecting cellular lines from cytotoxicity in the same manner. As the final representatives discussed here, polymers are tunable agents that can sustain the generation of new architectural platforms for antifungal drugs, the incorporation of nanoparticles, or the synthesis of nano-sized micellar systems. Their intrinsic mucoadhesive qualities led to antifungal activity for both synthetic and natural antimycotic agents, completing an authentic molecular map of antifungal mechanisms.

## Figures and Tables

**Table 1:** Local and systemic triggers defined by surface and molecular changes, involved in candidiasis development and pathogenesis.

Local Factors		
Factors	Surface and Molecular Effects	Ref.
Imbalances in salivary flow	A high number of *C. albicans* colonies were found in patients with xerostomia, which indicates alterations in normal microbiota. These changes are signaled by a modified pH in oral mucosa and the loss of antimicrobial proteins such as lysozyme, lactoperoxidase, immunoglobulin, histatins, and lactoferrin.	[27,43,44]
Dentures	Dentures decrease O_2_ supply at the epithelial level and reduce the salivary flow, creating a favorable acidic medium for yeast development. The fungi have an increased affinity for the roughened hydrophobic surfaces of acrylic resins of dentures, encouraging biofilm generation and the initiation of stomatitis. The adhesion process is promoted by a gradual replacement of interfacial water. Intimate mechanisms involved in biofilm formation are the roughness of the surface, hydrophobicity, and electrostatic nature of interactions prior to promoting protein adsorption and adhesion, Lifshitz–van der Waals forces, Brownian motion, and receptor–ligand binding.	[28,45,46]
Prosthesis	Dental implants favor the localization of *C. albicans* in the subgingival sulcus and the initiation of pathogenic periodontitis (peri-implantitis). Poor oral hygiene acts in the sense of promoting colonization of periodontal pathogens. The virulence of *C. albicans* is related to the activity of aspartyl proteinase as a promotor for adhesion. In addition, secretion of candidalysin will harm the epithelial cells and bind the epithelial growth factor ^1^ ErbB1 (Her1). The damage to the tissues of the peri-implant area is favored by metallopeptidase (95 kDa), targeting type I collagen, type IV collagen, fibronectin, and basement membrane.	[29]
Pre-existing oral pathologies	Denture stomatitis is promoted by modification in E-cadherin, collagen VII and fibronectin, combined with the presence of *C. albicans* colonies in the oral mucosa. Biopsy studies highlighted disorganization of the epithelial cells, with an irregular arrangement of keratinocytes and inflammatory phenomena in connective tissue.	[36,46]
Poor epithelial local defense	Reduced response of the host immune defense elements (Toll-like receptors, C-type lectin receptors, and ^2^ NOD-like receptors) induces dissemination of virulence factors, specific for *C. albicans* growth: dimorphism, adhesion, phenotypic switching, polymorphism, and secretion of hydrolytic enzymes such as lipases, phospholipases, and proteinases. A deficiency of epithelial antimicrobial peptides (AMP) was correlated with candidiasis development.	[31,47,48]
Oral dysbiosis	The oral microbiome covers a large number of microorganisms, up to 700 species, of which more than 60 are fungi species. A reduction in the number of native fungi that normally harbor the buccal mucosa was associated with the risk of developing oral infections. The interactions between fungal entities and bacteria such as streptococci favor the development of mixed biofilms and modulate the mechanisms implied in polymorphism and host immune response. The interactions of streptococci with *C. albicans* are made through the cell wall surface proteins of the Csh protein family and streptococcal surface proteins A and B. For its part, *C. albicans* supports the interactions through ^3^ ALS1, ^4^ ALS3, and ^5^ HWP1. Other interactions are governed by carbohydrate and extracellular polysaccharides, quorum-sensing molecules, and metabolic events. Dietary sucrose increases *C. albicans* virulence and the symbiotic relationship with streptococcus species.	[32,33,49]
Inhalator corticosteroids	The treatment with inhalator corticosteroids causes a poor epithelial local defense of the immune system and was thought to elevate salivary glucose levels as a substrate for fungi growth. Oral candidiasis development was dependent on dose and the device used in administration.	[34,50]
Smoking	Several theories consider the epithelial damage induced by smoking. In a more profound understanding, a concentration of 1–2 mg/mL of nicotine was found to assure the process of fungi cell multiplication, and it was correlated with an increase in HWP1 and ALS3 expression, implied in hyphae expansion and biofilm formation.	[16,35]
Carbohydrate-based diet	The intake of dietary and sugar-based foods represents a substrate for candidiasis development. High glucose levels in diabetic patients influence oral candidiasis development as well. Low blood glucose levels (~0.1%) stimulate hyphae growing as the invasive form of candidiasis. This is the result of the regulation of secreted aspartyl proteinases (SAP) genes under the signals of the ^6^ cAMP/PKA pathway and ^7^ MAP kinase cascade. MAP kinase pathway was recognized as a trigger for adhesion, invasion, and reorganization of the epithelial level.	[16,50,51]
**Systemic Factors**		
**Factors**	**Surface and Molecular Effects**	**Ref.**
Vitamin and mineral deficiencies	Candidiasis development can be promoted by vitamin A, B_6_, and B_12_ deficiency, iron deficiency, or a reduced level of essential fatty acids, folic acid, magnesium, selenium, or zinc. Concerning iron metabolism, it represents an essential element for cell differentiation, oxygen transport, and the normal activity of immune cells. The atrophy of oral mucosa and candidiasis were frequently discovered in anemic patients.	[16,37,52]
Metabolic disorders	Diabetic patients are highly predisposed to oral candidiasis, which can be seen as a pathological result of an accumulation of factors: poor oral hygiene, xerostomia, pH imbalances, increase in serum glucose levels, and poor epithelial local defense alike.	[38,53]
Menopause	A decrease in estrogen hormone levels in menopausal women can counteract the normal state of the oral mucosa. A hormonal change can induce a cascade of local effects involving the modification in salivary secretion, lysozyme decrease, poor local immune activity, and an increase in oxidative stress.	[39]
HIV immunodeficiency	Immunosuppression that can be quantified by a decreased number of CD4+ immune cells entails an increased risk of developing candidiasis. A decrease in histatins level contributes to the severity of the pathology.	[18,21,40]
Prolonged antibiotherapy	Administration of broad-spectrum antibiotics yields dysbiosis, affecting the normal oral flora and transforming the commensal microorganisms into pathogenic entities. Imbalances in oral microbiota were related to a decrease in salivary antibody content. Salivary proteins expressed as mucins, salivary IgA, cystatin S, basic proline-rich proteins, or statherins are implied in a dynamic process concerning adhesion/aggregation/clearance of fungal cells.	[21,54,55]
Immunosuppressive treatments	Immunosuppressive and cytotoxic treatments of malignancies promote a weakening of the immune system, with repercussions for epithelial cell defense. Resistance to antifungal therapy was observed due to the formation of biofilms with persistent *C. albicans* or *C. glabrata* cells.	[41,42,56]
COVID-19	COVID-19 induces immunosuppression by decreasing CD4+ and CD8+ T immune cells. In addition, candidiasis development in its invasive form has a multifactorial pattern, drawn by the presence of comorbidities (diabetes mellitus, pulmonary disorders, and malignancies) and concomitant treatments with immunosuppressants, corticosteroids, or antibiotics. Once more, a decrease in the salivary level of AMP was considered to be a robust marker for both superficial and intrusive infections.	[57,58]

Note: ^1^ ErbB1 (Her1) represents eukaryotic ribosome biogenesis protein, ^2^ NOD-like receptors—nucleotide-binding and oligomerization domain receptors, ^3^ ALS1—agglutinin-like sequence-1, ^4^ ALS3—agglutinin-like sequence-3, ^5^ HWP1—hyphae wall protein-1, ^6^ cAMP/PKA represents the cyclic adenosine monophosphate/protein kinase A pathway, and ^7^ MAP kinase—mitogen-activated protein kinase.

**Table 2:** Classic antifungal drugs, their mechanisms of action, and major drawbacks in terms of fungal resistance to antifungal therapy.

Therapeutic Class	Antifungal Drugs— Main Compounds	Mechanisms of Action	Mechanisms of Resistance	Drug-Related Drawbacks	Ref.
Polyenes	Nystatin Amphotericin B Natamycin	Target the ergosterol sites and disrupt the cellular membrane, promoting fungicidal effects. Fungicidal action is enhanced by inducing oxidative stress.	Modification of enzymes with catalytic activity (C_8_-sterol isomerase and Δ_5,6_-desaturase), implied in ergosterol biosynthesis, via ERG2 and ERG3 gene alteration. Increase catalase function and deviate ROS generation.	Nystatin is used only for its local effect, without systemic absorption. It has an unpleasant taste in buccal administration.	[16,20,107,116]
				Amphotericin B is nephrotoxic and preferred as second-line therapy.	
				Poor bioavailability.	
Azoles	Clotrimazole Miconazole Fluconazole Posaconazole Itraconazole Voriconazole	Inhibition of cytochrome P450 enzymes implied in the biosynthesis of ergosterol from lanosterol, namely 14-α demethylase. The global effect is based on morphological alteration of fungi cells and inhibition of fungi growth.	Overexpression of CDR1 and CDR2 of the ATP-binding cassette superfamily, and MDR1 (major facilitator superfamily) genes. Use of efflux pumps by regulation of Tac1 and Mrr1 ATP binding cassette genes. Structural modification of demethylase enzyme. Overexpression of ERG11 gene. Cross-resistance with polyenes via ERG3 gene alteration. Drug engulfment into a complex glucan-based matrix of the biofilm.	Poor bioavailability, and buccal delivery of azoles is less efficient (multiple dosings and short local retention).	[113,117,118]
Echinocandins	Micafungin Caspofungin Anidulafungin	Fungicidal effects induced via inhibition of β (1–3) glucan synthase. Drug molecules target cell wall proteins implied in β (1–3) glucan synthesis.	Mutation of FKS1 gene specific for the catalytic unit of glucan synthase. Increased chitin synthesis. Drug engulfment into a complex glucan-based matrix of the biofilm. Imposition of adaptive mechanisms of fungi cells via Hsp90, cell wall integrity pathway, or high-osmolarity glycerol pathway.	Poor bioavailability, and the intravenous route (i.v.) is only accepted for systemic treatment.	[8,119]
Pyrimidines antimetabolite-like	5-fluorocytosine	Cytosine permease entraps drug molecules into the cells. 5-fluorocytosine is bio transformed in 5-fluorouracil in the presence of cytosine deaminase. The new compound alters RNA synthesis and consequently protein synthesis. A second conversion through fluoro-deoxyuridylic acid determines DNA alteration.	Mutations in cytosine permease and cytosine deaminase enzymes.	Drug monitoring is mandatory for oral and i.v. route to avoid immunosuppression and hepatotoxicity.	[107,119,120]

## Data Availability

Not applicable.
